# Maternal protein restriction affects fetal ovary development in sheep

**DOI:** 10.1530/RAF-20-0073

**Published:** 2021-06-17

**Authors:** Chinwe U Nwachukwu, Kathryn J Woad, Nicole Barnes, David S Gardner, Robert S Robinson

**Affiliations:** 1 School of Veterinary Medicine and Science, Sutton Bonington campus, The University of Nottingham, Loughborough, Leicestershire, UK; 2 Department of Agricultural Science, School of Agriculture and Vocational Studies, Alvan Ikoku Federal College of Education, Owerri, Imo State, Nigeria; 3 Medivet Oxted, Oxted, UK

**Keywords:** sheep, fetal ovary, germ cells, maternal protein

## Abstract

Maternal malnutrition has important developmental consequences for the foetus. Indeed, adverse fetal ovarian development could have lifelong impact, with potentially reduced ovarian reserve and fertility of the offspring. This study investigated the effect of maternal protein restriction on germ cell and blood vessel development in the fetal sheep ovary. Ewes were fed control (*n* = 7) or low protein (*n* = 8) diets (17.0 g vs 8.7 g crude protein/MJ metabolizable energy) from conception to day 65 of gestation (gd65). On gd65, fetal ovaries were subjected to histological and immunohistochemical analysis to quantify germ cells (OCT4, VASA, DAZL), proliferation (Ki67), apoptosis (caspase 3) and vascularisation (CD31). Protein restriction reduced the fetal ovary weight (*P* < 0.05) but had no effect on fetal weight (*P* > 0.05). The density of germ cells was unaffected by maternal diet (*P* > 0.05). In the ovarian cortex, OCT4+ve cells were more abundant than DAZL+ve (*P* < 0.001) and VASA+ve cells (*P* < 0.001). The numbers, density and estimated total weight of OCT4, DAZL, and VASA+ve cells within the ovigerous cords were similar in both dietary groups (*P* > 0.05). Similarly, maternal protein restriction had no effect on germ cell proliferation or apoptotic indices (*P* > 0.05) and the number, area and perimeter of medullary blood vessels and degree of microvascularisation in the cortex (*P* > 0.05). In conclusion, maternal protein restriction decreased ovarian weight despite not affecting germ cell developmental progress, proliferation, apoptosis, or ovarian vascularity. This suggests that reduced maternal protein has the potential to regulate ovarian development in the offspring.

**Lay summary:**

Variations in a mother’s diet during pregnancy can influence her offspring’s growth and might cause fertility problems in the offspring in later life. We investigated whether reducing the protein fed to sheep during early pregnancy affects their daughters’ ovaries. We then compared our findings to the offspring of sheep on a complete diet. We measured ovary size and estimated the number of germ cells (cells that become eggs) they contained. We used cell markers to assess potential changes in the pattern of germ cell growth, division, and death, and how the ovarian blood supply had developed. We found that protein restriction reduced ovary size. However, the pattern of germ cell development, growth, or death was not altered by poor diet and blood vessels were also unaffected. This suggests that maternal diet can change ovarian development by an unknown mechanism and might reduce future fertility in their offspring.

## Introduction

Malnutrition poses a significant health burden, with over 400 million adults suffering from underweight worldwide (WHO 2020*a*). Even as the rates of overweight and obesity continue to increase in many countries, undernutrition and poor-quality nutrition remain a serious public health concern. Furthermore, since maternal malnutrition is associated with adverse pregnancy and birth outcomes, improved maternal nutrition is acknowledged as a fundamental global health target (WHO 2020*b*).

Maternal undernutrition can also have detrimental impacts on ovarian development in the offspring, with the potential for lasting influence on fertility in adulthood (Meikle & Westberg 2001, Long *et al.* 2010). Given that the ovary has limited capacity for postnatal oogenesis, any alteration in the establishment of the ovarian reserve represents a lifetime deficit that cannot be recouped (Findlay *et al.* 2015).

Gestational dietary restriction has been shown to impact ovarian development in a range of species including pigs (Sui *et al.* 2014*b*), sheep (Rae *et al.* 2001, Lea *et al.* 2006), cattle (Mossa *et al.* 2013) and rodents (Sloboda *et al.* 2009, Bernal *et al.* 2010). These dietary regimes have included reduced total rations, calories, or protein and have varied widely by the degree, timing and duration of restriction imposed, plus time point of assessment. The provision of inadequate protein in the maternal diet is thought to have a greater impact upon fetal development than a balanced reduction in energy intake, as the deficit in amino acids requires a more substantial shift in maternal metabolism (Dunford *et al.* 2014).

Adverse outcomes in the malnourished foetus have included reduced ovarian mass (Rae *et al.* 2001), altered fetal germ cell numbers and dysregulated entry into meiosis (Borwick *et al.* 1997). Whilst in adulthood, animals which experienced gestational nutrient restriction had reductions in antral follicle count (Mossa *et al.* 2013), oestrous cycle length (Long *et al.* 2010), progesterone production (Sloboda *et al.* 2009) and number of offspring (Meikle & Westberg 2001, Long *et al.* 2010) plus an advanced pubertal onset (Sloboda *et al.*, 2009). In addition, in girls with a low birth rate, growth restraint resulted in a reduced ovulation rate at adolescence (Ibáñez *et al.* 2002). In contrast, others have argued that there is little good evidence that prenatal undernutrition is detrimental to adult fertility (Gardner *et al.* 2009), whilst recent evidence suggests that gestational undernutrition can even improve fertility markers (Smith *et al.* 2019).

Whilst the underlying mechanisms through which maternal undernutrition programs offspring reproduction remain unclear, disrupted ovarian development has been associated with alterations in ovarian gene expression and regulatory microRNA abundance (Sui *et al.* 2014*a*,b), differential DNA methylation (Sui *et al.* 2014*b*), a shift in the balance of cell proliferation to apoptosis (Lea *et al.* 2006) and diminished oxidative stress response (Murdoch *et al.* 2003, Bernal *et al.* 2010), including in response to restricted protein (Sui *et al.* 2014*a*,b).

The establishment of an appropriate vasculature is critical to an organ’s function and has been shown to be sensitive to nutritional insults. Indeed, the present study utilised fetal ovarian tissue from the same animals where maternal protein restriction resulted in a microvascular compromise in the fetal kidney, reducing endothelial cell number and angiogenic factor expression (Dunford *et al.* 2014). Similarly, in the rat, maternal protein restriction reduced fetal pancreatic vascularisation (Boujendar *et al.* 2003). Periods of maternal underfeeding also increased the expression of apoptosis regulatory factors Bax and Mcl1 in endothelial and perivascular cells of the fetal sheep ovary (Lea *et al.* 2006) and reduced the vascular proliferation index (Grazul-Bilska *et al.* 2009).

Therefore, our study tested the hypothesis that maternal protein restriction would adversely affect germ cell development and the vascularisation of thefetal sheep ovary at 65 days of gestation. The objectives were to (1) examinefetal ovarian morphology and develop by immunolocalising specific markers of germ cell development, (2) determine the rate of proliferation and apoptosis, and (3) assess the degree of vascularisation within the wholefetal ovary.

## Materials and methods

### Animal study

All procedures were performed in accordance with the United Kingdom (UK) Animals (Scientific Procedures) Act, 1986 (Amended Regulations 2013) and were approved by the Animal Welfare and Ethical Review Board of the University of Nottingham.

Scottish Blackface ewes (*n* = 31) carrying only twins were randomly assigned to groups and fed either a control diet providing adequate protein (180 g/kg crude protein), or a low protein diet (80g/kg crude protein) (AFRC 1993). The diets were isocaloric as-fed, with an effective protein level of either 17.0 g crude protein/MJ metabolizable energy (control protein; CP, *n* = 7) or 8.7 g crude protein/MJ metabolizable energy (low protein: LP, *n*  = 8). The diet was formulated by Dr Stewart Rhind, Macaulay Institute, Aberdeen and as previously described (Dunford *et al.* 2014). The formulated diet was fed from the day of AI to day 65 of gestation (gd65) when the sheep were sacrificed by barbiturate overdose, slaughtered and fetal tissues were collected. One twin (male or female) was subjected to a whole animal vascular cast (Dunford *et al.* 2014). If the other twin was female, then both ovaries were collected (*n* = 15) and weighed. One ovary was selected at random and fixed in Bouin’s for 6 h, then paraffin-embedded according to standard procedures and used for blinded histological and immunohistochemical analysis.

### Histological and immunohistochemical analysis

The tissue blocks were serially sectioned throughout at 5 µm (RM 2255 microtome, Leica Microsystems). Two sequential tissue sections were mounted onto each poly-L-lysine coated glass slide (Fisher Scientific, Loughborough, UK). The mean number of sections per ovary was 252.

Haematoxylin and eosin staining was performed on every 20th slide (both sections) commencing at slide 1 for morphological assessment of the fetal ovaries and identification of germ cells within the ovigerous cords as previously described (McNatty *et al.* 2000, Sawyer *et al.* 2002). The identification of the basement membranes surrounding the ovigerous cords and vasculature was performed with periodic acid-Schiff (PAS) staining (Reynolds & Redmer 1992) on every 40th slide, commencing at slide 2. Next, immunohistochemistry to identify and quantify germ cells (OCT4 (PGC/oogonia), DAZL (early oogonia), and VASA (early oogonia/primary oocyte)), proliferation (Ki67), apoptosis (caspase 3) and vasculature (CD31) (see Table 1 for details). Each marker was analysed every 20th slide (both sections) commencing at slides 3, 4, 5, 6, 7 and 8, respectively. Briefly, all sections were rehydrated using standard protocols prior to antigen retrieval by boiling in 10 mM citrate buffer of pH 6.0 for 10 min. This was followed by a peroxidase block step with 3% (v/v) hydrogen peroxide in methanol, then an antigen block with either 2% (v/v) normal goat or horse serum. The sections were incubated with primary antibody overnight at 4°C, while negative control slides had an equivalent concentration of rabbit or mouse IgG (Sigma–Aldrich). On the next day, primary antibodies were detected using the ABC method (Vector Labs, Peterborough, UK) as per the manufacturer’s instructions. DAB substrate (Vector) was used to visualise the antibody complexes. The sections were subsequently counterstained with haematoxylin for 20 s, then dehydrated and mounted in DPX mountant.

**Table 1:** Details of antibodies used for immunohistochemistry analysis.

Antigen	Function/marker	Source	Host species	Dilution factor	Final concentration (µg/mL)
Oct4	Pluripotency markers (e.g. PGCs)	ab19857; Abcam	Rabbit, polyclonal	1:250	4
DAZL	Germ cell marker	ab34139; Abcam	Rabbit, polyclonal	1:250	4
VASA	Germ cell marker	ab13840; Abcam	Rabbit, polyclonal	1:300	3.3
Ki67	Cellular proliferation	VP-K452; Vector Labs	Mouse, monoclonal	1:80	6
Caspase 3	Cellular apoptosis	#9579; Cell Signalling	Rabbit, polyclonal	1:200	3
CD31	Vascular endothelial cells	ABIN1582260; Abbiotec	Rabbit, polyclonal	1:300	3.3

PGC, primordial germ cell

### Image analysis and calculations

**Figure 1:**
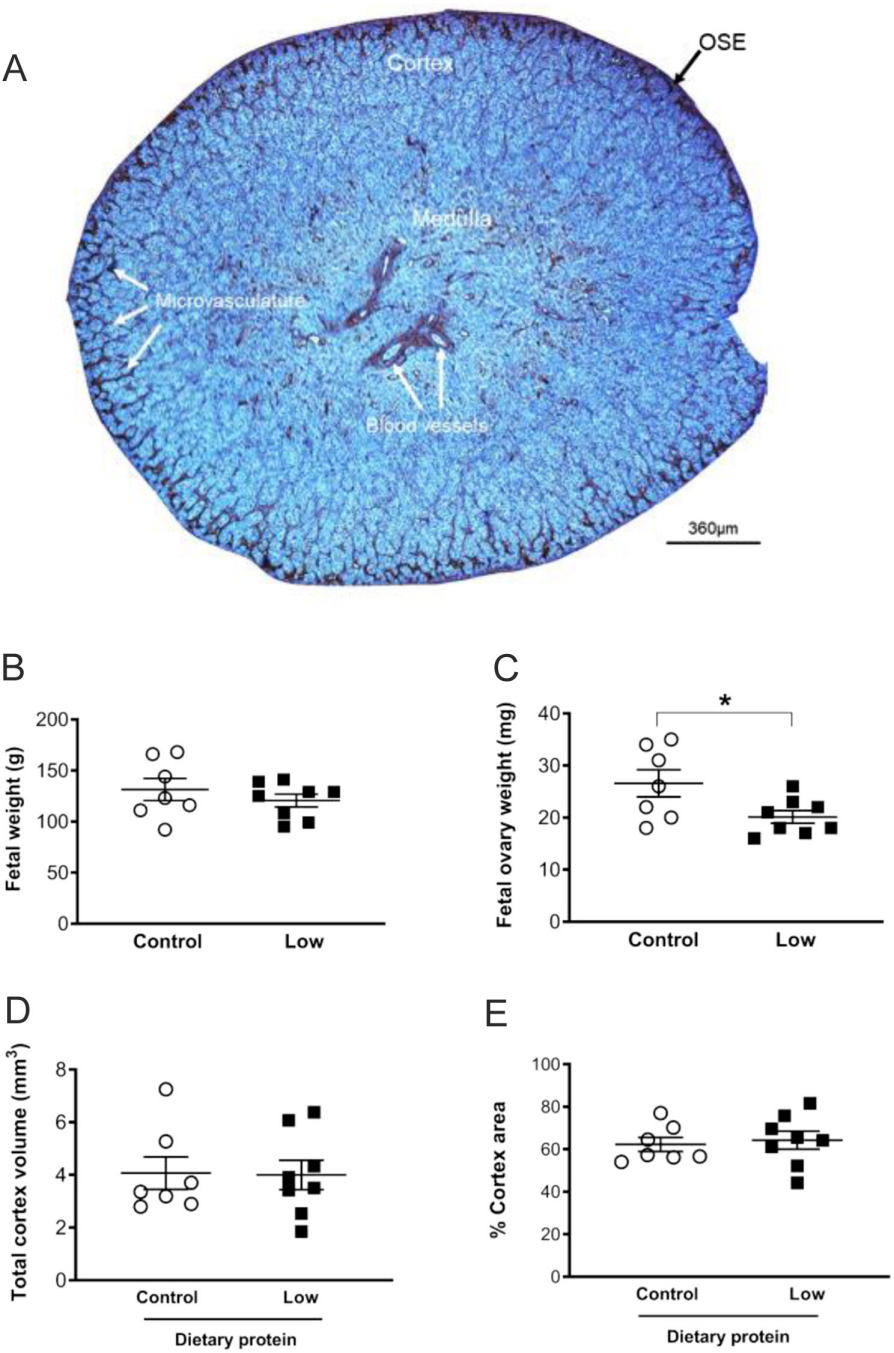
The effect of maternal dietary protein restriction on fetal weight and fetal ovary characteristics of sheep on gestation day 65. (A) Low magnification (5×) image for CD31 immunostained fetal ovary highlights the vasculature (brown) and enables the visualization of the ovarian surface epithelium (OSE), cortex and medulla regions. The scale bar is 360 µm. Quantification of (B) fetal weight; (C) fetal ovary weight (both ovaries); (D) total cortex volume; (E) percentage area of cortex. Data are mean ± s.e.m. with * (*P* < 0.05) vs control. Fetal ovarian weight was decreased in the low protein dietary group (*n* = 8) compared with the control group (*n* = 7).

The proportion of the fetal ovarian area occupied by the cortex was determined using the CD31-immunostained sections, as there was a clear demarcation between the medulla and cortex regions (Fig. 1). For this, complete cross-section images from every CD31-immunostained section were captured using a light microscope with a 5× objective (DM500B, Leica Microsystems) and camera (DFC350FX, Leica Microsystems) which was repeated across the whole fetal ovary. Image analysis was conducted using Image ProPlus 6.3 (Media Cybernetics, Marlow, UK). Next, the area of the whole fetal ovary and its medulla were measured by drawing around the section edge and medulla-cortex demarcation, respectively. From this, the total area and proportion occupied by the cortex were calculated. The total ovarian volume (V_(0)_) was then estimated using the Cavalieri principle (Gundersen & Jensen 1987, Smith *et al.* 1993). Namely, the mean area of each section was multiplied by the thickness of the section (5 µm) and by the total number of sections per fetal ovary. The total medulla volume was calculated in a similar manner and this was subtracted from the V_(0)_ to yield the estimated total cortex volume (V_(c)_). For the other histological and immunohistochemical parameters, four images per section (total of eight images per slide) were captured randomly across the cortex region of the section.

#### Histological analysis

Germ cells within ovigerous cords were identified based on their H&E-stained histological appearance and were manually counted using the pointer function (Image ProPlus 6.3). The mean number of germ cells per section was calculated from the four fields of view (only from the cortex). Germ cell density was calculated by dividing the number of germ cells by the area of each field of view. The estimated total number of germ cells was then calculated as follows: (1) the mean germ cell diameter (14.2 µm) was determined (from >100 cells across different animals, with no difference between dietary groups). This was then converted to an area of 158.4 µm^2^ using the area of a circle (πr^2^). Next, it was assumed that the germ cells were spherical, and the volume was calculated as 1499 µm^3^ using 4/3 𝜋𝑟^3^. (2) The percentage area occupied by germ cells was then calculated by multiplying germ cell count by the mean area of each germ cell and then dividing this by the total field of view area. (3) The estimated total number of germ cells (N) in a single ovary was calculated based on Smith *et al.* (1993) and shown in Equation 1. This number was multiplied by 2 to obtain the total number of germ cells in both ovaries.


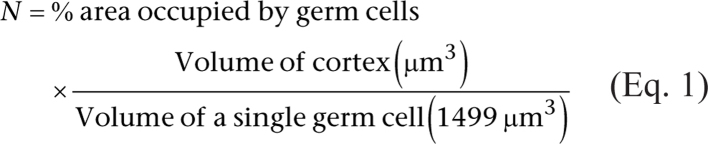


#### Periodic acid-Schiff staining

The area and percentage area of periodic acid-Schiff (PAS) staining was determined by manually selecting the magenta PAS stain associated with the ovigerous cords using the 'Area of Interest' tool in Image ProPlus 6.3 and the count/size tab.

#### Immunohistochemical staining

For each germ cell marker (OCT4+ve; DAZL+ve; VASA+ve), four fields of view were captured across each section using a light microscope with a 5× objective (DM500B, Leica Microsystems) and camera (DFC350FX, Leica Microsystems). Germ cells (total number within ovigerous cords) were manually counted using the pointer tool (Image ProPlus) for each image. The mean germ cell count was calculated from the four fields of view across each section. This count was then repeated for every stained section across the whole fetal ovary. This was used to determine an overall mean count, density, and total number of germ cells. The ratio between the different germ cell markers was also calculated. The proliferation index was calculated as the proportion of all germ cells (within the ovigerous cord) which were Ki67+ve, while the apoptotic index was calculated as the proportion of germ cells which were caspase 3+ve.

#### Vascularisation analysis

The CD31 immunohistochemical staining was performed to assess two principle components of fetal ovary vascularisation: (1) the degree of microvascularisation within the ovarian cortex, and (2) the area occupied by the larger blood vessels within the ovarian medulla (Fig. 1A).

Degree of microvascularisation in the cortex: for each field of view (*n* = 4/section), the colour select tool was then used to identify the brown colour (CD31 staining) and determine the percentage area of CD31 staining within the microvasculature region of the cortex. This was repeated for each section, from which an overall mean was calculated.

Size and dimensions of blood vessels in the medulla: the whole medulla region was captured under 40× objective. The pointer tool was used to draw around the lumen of each vessel stained by CD31. Then, Image ProPlus determined the number of blood vessel cross sections, lumen area and perimeter. This was repeated for each CD31-stained section per ovary, from which an overall mean was calculated.

### Statistical analysis

All data were checked for normality and heterogeneity of variance before statistical analysis. GenStat was used to analyse the statistical significance (*P* < 0.05) of differences. The effect of protein restriction on all data points was analysed by the Student’s (unpaired) *t*-test using GenStat. Results were expressed as mean ± s.e.m.

## Results

### Organ weights and structure

Meanfetal weight at gd65 was not different between the two dietary groups (*P* > 0.05; Fig. 1B) butfetal ovary weight was reduced by maternal protein restriction (*P* < 0.05; 20 ± 1.2 mg vs 26 ± 2.6 mg; Fig. 1C). However, the calculated ovarian volumes were not different between dietary groups (6.6 ± 0.9 mm^3^ (control) vs 6.3 ± 0.8 mm^3^ (LP)). In addition, the percentage area occupied by the cortex and the estimated total volume of the cortex were unaffected by maternal diet (*P* > 0.05; Fig. 1D and E).

**Figure 2:**
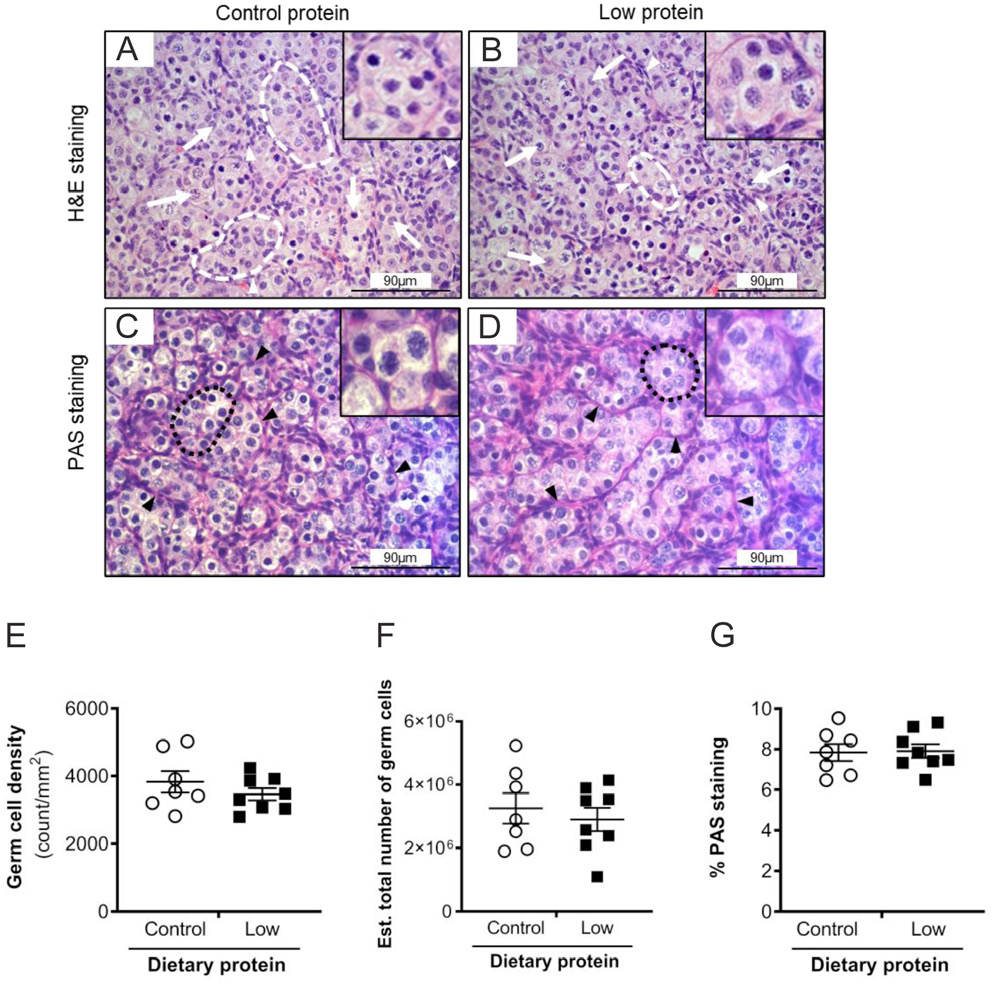
The effect of maternal dietary protein restriction on germ cell and basement membrane morphology on gestation day 65. Representative images of H&E stained (A,B) and PAS-stained (C,D) sections ofetal ovaries following (A,C) control and (B,D) low protein diets. In A and B: germ cells (white arrows) were organised into ovigerous cords enveloped by a basement membrane (white dashed line) and stromal cells (white arrowheads). In C and D: PAS staining (magenta) was associated with the basement membrane (black arrowheads) surrounding ovigerous cords (black dotted line); scale bars = 90 µm. Graphs show (E) density (count/mm^2^), (F) estimated total number of germ cells and (G) percentage area of PAS staining. Data are mean ± s.e.m. The estimated total weight of germ cells was decreased after maternal protein restriction (*n* = 8) vs control (*n* = 7).

Histologically, germ cells were identified as near circular structures with a centrally located nucleus that was larger than the other ovarian cells. They were observed in clusters (egg nests) with a surrounding basement membrane (Fig. 2A and B). However, there were no obvious morphological differences in egg nests or the whole ovary structure between maternal diets (Fig. 2A and B). Using periodic acid-Schiff staining, the basement membrane was evident in the vascular components and particularly intense in the envelopes that surrounded the egg nests within the ovarian cortex (Fig. 2C and D) and this was present in both dietary groups.

### Ovarian germ cells

The mean density of germ cells within the cortex was unaffected by diet (Fig. 2E). Similarly, the estimated total number of germ cells in both ovaries was approximately 4 million but this was unaffected by diet (*P* > 0.05; Fig. 2F). In addition, there was no effect of diet on the percentage area of PAS staining (*P* > 0.05; Fig. 2G).

**Figure 3:**
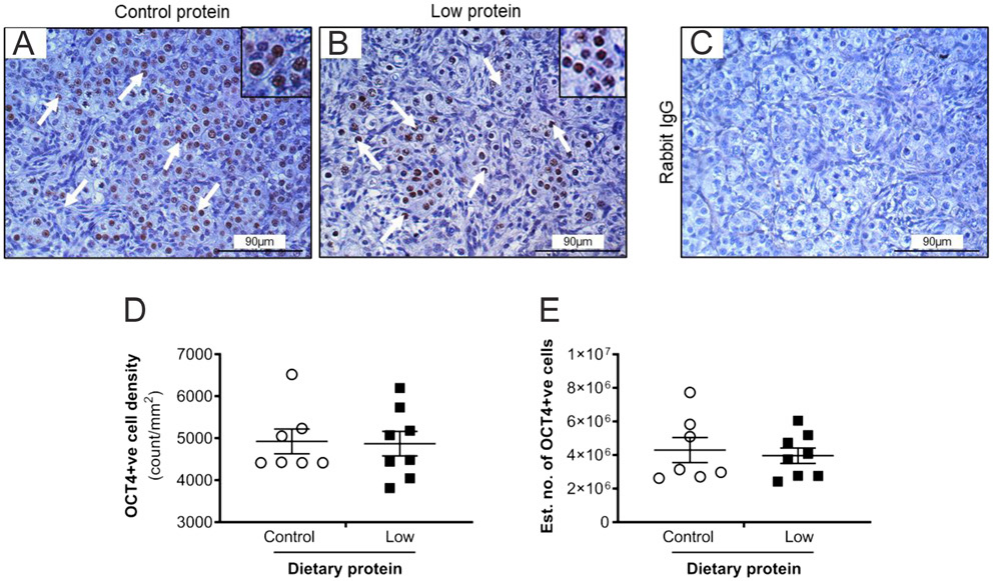
The effect of maternal dietary protein restriction on OCT4+ve germ cell development on gestation day 65. Representative images ofetal ovaries from sheep fed (A) control protein); (B) low protein diets, while (C) shows the absence of staining with control rabbit IgG. The insets showing higher magnification images. Photomicrographs show germ cells (arrows) organised into ovigerous cords enveloped by a basement membrane. Scale bars = 90 µm. Graphs show that (D) density and (E) estimated number of OCT4+ve cells were unaffected by diet. Data are mean ± s.e.m. from control (*n* = 7) and low protein (*n* = 8) diets.

**Figure 4:**
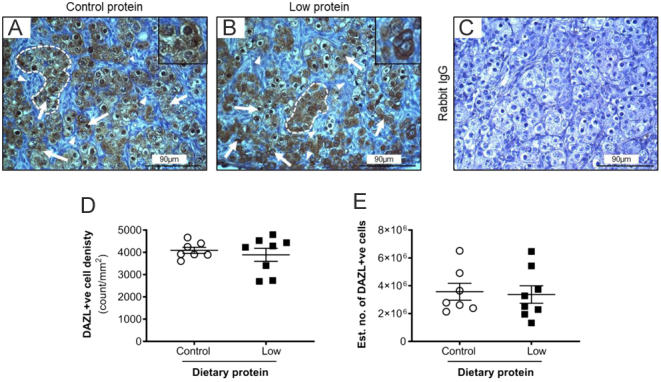
The effect of maternal dietary protein restriction on DAZL+ve germ cell development on gestation day 65. Representative images ofetal ovaries from sheep fed (A) control protein; (B) low protein diets. (C) shows the absence of staining with rabbit IgG. Photomicrographs show germ cells (GC, arrows) organised into ovigerous cords (dashed line) with insets showing higher magnification. DAZL-ve stromal cells were also observed (arrowheads). Scale bars = 90 µm. Graphs show that the (D) density, and (E) estimated number of DAZL+ve cells were unaffected by diet. Data are mean ± s.e.m. from control (*n* = 7) and low protein (*n* = 8) diets.

**Figure 5:**
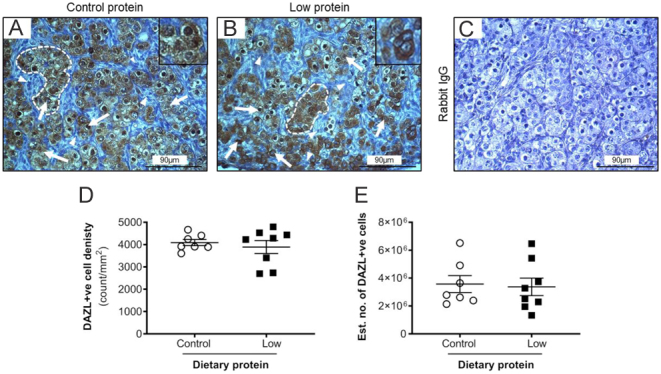
The effect of maternal dietary protein restriction on VASA+ve germ cell development on gestation day 65. Representative images ofetal ovaries from sheep fed (A) control protein; (B) low protein diets. Photomicrographs show germ cells (arrows) organised into ovigerous cords. VASA-ve germ cells were also observed. (C) shows the absence of staining with rabbit IgG. Insets show higher magnification. Scale bars = 90 µm. Graphs show that the (D) density and (E) estimated number of VASA+ve cells were unaffected by diet. Data are mean ± s.e.m. from control (*n* = 7) and low protein (*n* = 8) diets.

The next step was to determine whether the development of germ cells was affected by maternal diet. We chose to investigate this by quantifying immunohistochemistry of OCT4, VASA and DAZL (Figs 3, 4, 5). OCT4+ve staining was observed in the nuclei of germ cells, predominantly localised in the cortex proximal to the ovarian surface epithelium (Fig. 3A and B). Furthermore, the OCT4+ve cells differed in their size across the section, with those near to the ovarian surface epithelium, appearing to be smaller and more densely packed than those near to the medulla. Staining was absent in the immunohistochemical controls (Fig. 3C). OCT4-ve cells were also observed within the ovigerous cords which were small, oval-shaped, and closely associated with OCT4+ve cells. In each ovigerous cord cross-section, there were one or two OCT-ve cells which we interpreted as pre-granulosa cells.

Abundant DAZL+ve staining was detected within the germ cells of the ovigerous cords (Fig. 4A and B). DAZL staining was particularly intense in the nucleus but was also present in the cytoplasm. A small proportion of germ cells were DAZL−ve but there was no apparent difference with DAZL+ve cells with respect to diameter, position relative to the ovarian surface epithelium or proximity to other neighbouring germ cells. The immunohistochemical controls displayed no brown staining (Fig. 4C). VASA immunostaining was detected in the cytoplasm of germ cells (Fig. 5A and B) and its intensity appeared greater in those cells with a larger diameter. The majority of germ cells within a particular ovigerous cord were VASA+ve. In several germ cells, a distinct compact perinuclear structure, putatively identified as the Balbiani body (Pepling *et al.* 2007), showed accentuated VASA immunostaining (Fig. 5A and B). There was no effect (*P* > 0.05) of diet on the density (count/mm^2^) or estimated total number of OCT4+ve (Fig. 3D and E), DAZL+ve (Fig. 4D and E) and VASA+ve cells (Fig. 5D and E) in thefetal sheep ovaries on gd65. Across both diets, OCT4+ve cells were more abundant than DAZL+ve cells (*P* < 0.001), which, in turn, were two-fold greater in abundance than VASA+ve cells (*P* < 0.001).

Taken together, the relative abundance of major developmental cell markers in thefetal ovaries at gd65 indicated that, as expected, the majority of the germ cells were in the early stages of oogenesis. Importantly, however, the relative abundance of the germ cell markers was not significantly affected by maternal diet (*P* > 0.05). Nevertheless, similar ovarian reserve capacity may belie differences in rates of relative proliferative activity or programmed cell death, each of which are important biological processes during the development of the full complement of ovarian reserve.

### Ovarian germ cell proliferation and vascularisation

**Figure 6:**
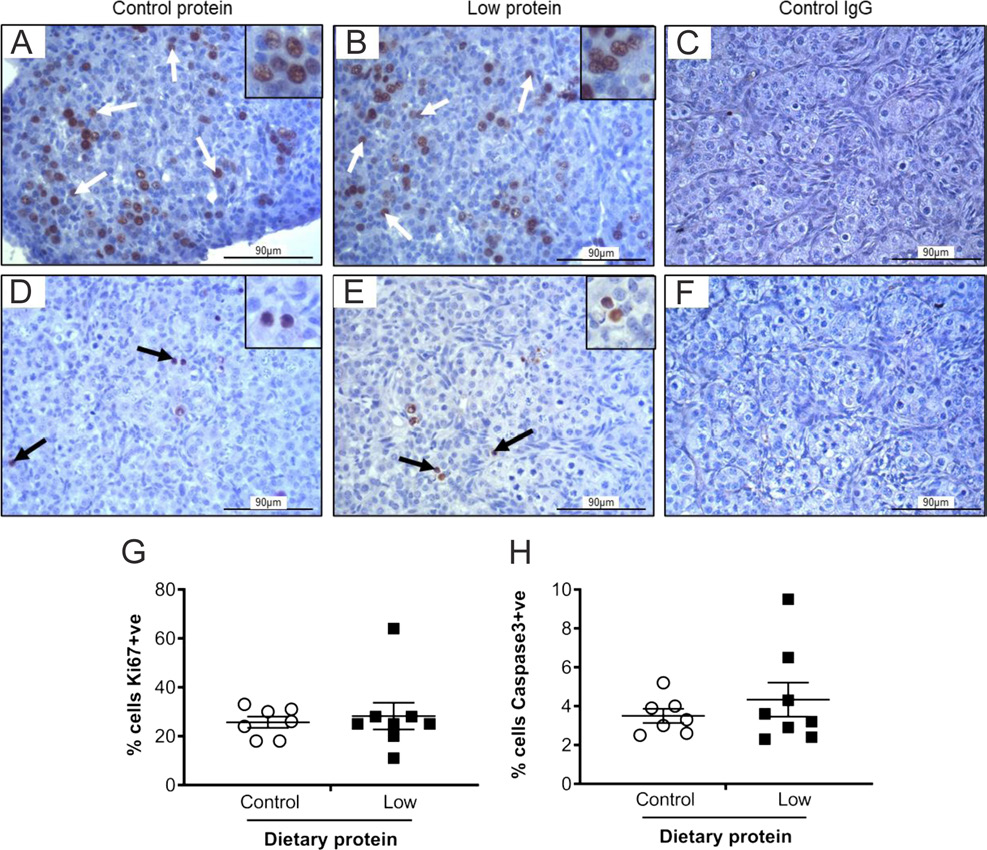
The effect of maternal dietary protein restriction on cell proliferation (Ki67; A,B,C) and apoptosis (caspase 3 staining; D,E,F) on gestation day 65. Representative images ofetal ovaries from sheep fed control (A,D) or low protein (B,E) diets. (C, F) show the absence of staining with control IgG. Positive staining is indicated by arrows with insets showing images at a higher magnification. Scale bars = 90 µm. Graphs show the proliferation (G) and apoptotic (H) indices which were unaffected by diet. Data are mean ± s.e.m. from control (*n* = 7) and low protein (*n* = 8) diets.

We, therefore, investigated whether rates of germ cell proliferation (Ki67) or apoptosis (caspase 3) were affected by maternal diet. There was an extensive number of cells in the ovarian cortex that were positive for Ki67, suggesting a high rate of proliferation in thefetal ovary at this stage of gestation. This was particularly evident in the region nearest the ovarian surface epithelium (Fig. 6A and B), where the proliferation index was quantified at 25–30%. Nevertheless, there was no effect of maternal diet (*P* > 0.05; Fig. 6G). Caspase 3 was immunolocalised to the nucleus of a small proportion of germ cells (Fig. 6D and E), with a mean apoptotic index of 3.9%. Again, there was no effect of diet on the apoptotic index (*P* > 0.05; Fig. 6H). Caspase 3 and Ki67 staining were observed in other cell types in thefetal ovary but this was sporadic and was not quantified.

**Figure 7:**
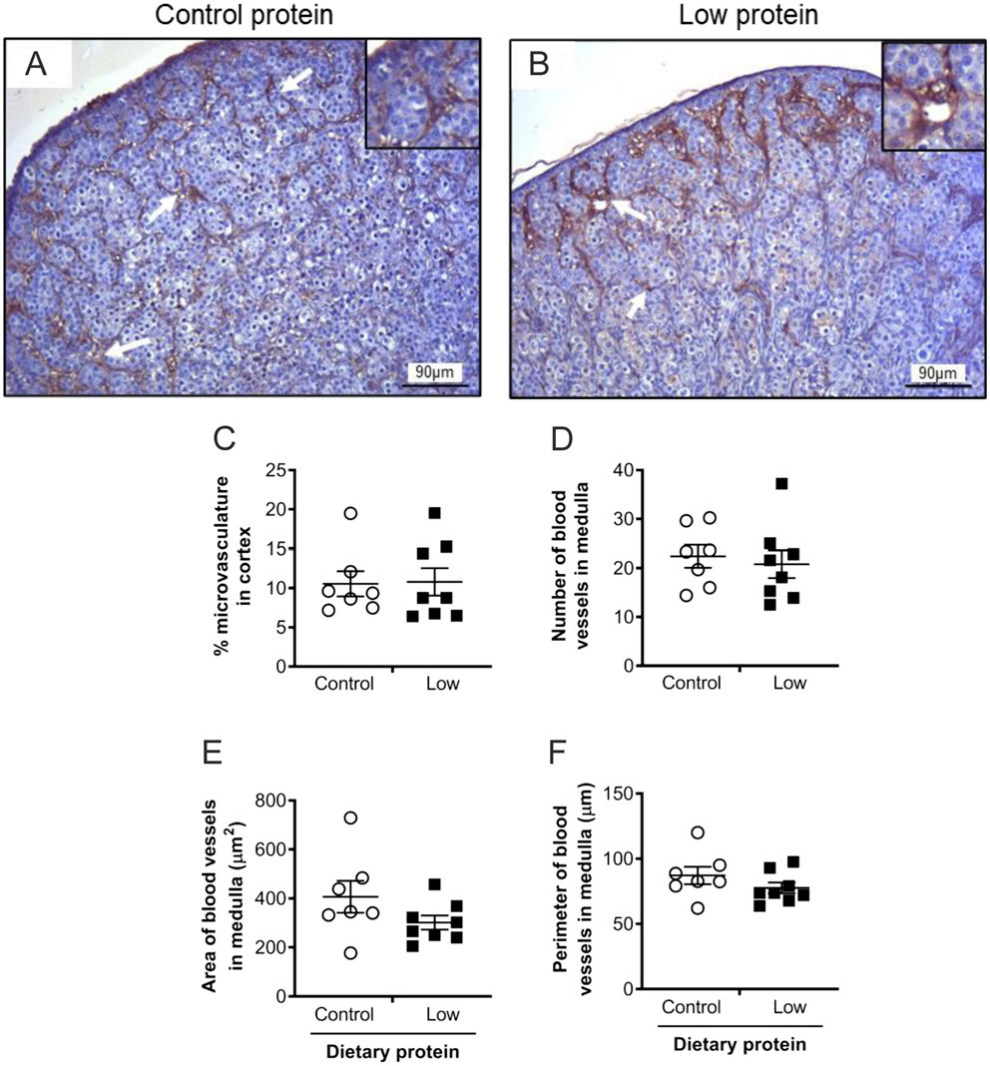
The effect of maternal dietary protein restriction on thefetal ovarian vascularisation on gestation day 65, as determined by CD31 immunostaining. Representative images ofetal ovaries from sheep fed (A) control protein (*n* = 7); (B) low protein (*n* = 8) diets; microvessels (arrows) ofetal ovarian cortex and medulla are shown. Photomicrographs show CD31-positive endothelial cells highlighting the microvascularised area of thefetal ovarian cortex (arrows). Scale bars = 90 µm. Graphs show (C) the percentage microvascularised area of the cortex, and (D) number, (E) area of lumen and (F) perimeter of blood vessels in the medulla. There was no effect of diet. Data are mean ± s.e.m.

The degree ofetal ovarian vascularisation at gd65 was determined by CD31 immunohistochemistry. The vascularisation analysis was split into two principle components: (1) microvasculature in the ovarian cortex (Fig. 7A and B) and (2) the larger blood vessels within the ovarian medulla (Fig. 1A). The numerous capillaries within the ovarian cortex were long, thin, and thread-like, suggestive of a complex microvasculature (Fig. 7A and B). The percentage area of the cortex that was microvascularised was not significantly different between the diets (*P* > 0.05; Fig. 7C). The blood vessels within the medulla were fewer in number, but much greater in size, with a clear lumen and a more extensive vessel wall, indicating larger arterioles and/or venules and larger capillaries (Fig. 1A). The average number, lumen area and perimeter of the blood vessels within the ovarian medulla were not altered by maternal diet (*P* > 0.05; Fig. 7D and F).

## Discussion

This study investigated whether a 50% reduction in maternal gestational protein from conception adversely affected the structure and development of the sheepfetal ovary at gd65 (i.e. 0.44 term in sheep). Maternal protein restriction reducedfetal ovary weight by approximately 30%. In the same fetuses, the relative size (organ weight/fetal body weight) of other organs (e.g. heart, kidney and liver) were unaffected (Dunford *et al.* 2014). Conversely, previous studies showed that maternal global undernutrition (100% vs 50% energy requirement) did not alter meanfetal ovarian mass on gd60 or gd110 (Rae *et al.* 2001), while underfeeding has counter-intuitively led to increasedfetal ovarian weight (Borwick *et al.* 1997, Osgerby *et al.* 2002).

### Germ cell development

The morphological characteristics and quantification of germ cells were determined using several endpoints (H&E in conjunction with specific germ markers (OCT4, DAZL, VASA)). The expression pattern of each marker on gd65 was broadly in agreement with previous studies (i.e. McNatty *et al.* 1994, Sawyer *et al.* 2002, Anderson *et al.* 2007) with the germ cells localised to ovigerous cords that were especially prominent in the cortex. The expression of OCT4, DAZL and VASA changes as the germ cell develops in thefetal ovary (Hummitzsch *et al.* 2013, 2015). In the present study, there was an overlap in the expression of these markers, but there were some distinct differences. For example, OCT4 was detected in germ cells of all sizes, while DAZL and VASA were more limited to the larger germ cells within the ovigerous cord. As expected at this gestational stage, OCT4 was the most abundantly expressed germ cell marker which would indicate that these cells were primordial germ cells or early oogonia (Anderson *et al.* 2007). To further support this, they were predominantly located in the periphery of the cortex. In contrast, DAZL+ve and VASA+ve cells were more evident in the cortex region nearer to the medulla. It is most likely that these cells are more developed and were either oogonia or early primary oocytes.

In the present study, maternal protein restriction markedly reduced thefetal ovarian weight by 30%, but there was no apparent difference in the density or estimated total number of germ cells. The latter point agrees with previous studies that showed maternal undernutrition (50–60% energy requirement), did not alter the total number of germ cells at a similar stage of gestation (Rae *et al.* 2001, Smith *et al.* 2019). However, this was associated with a reduction in the number of germ cells entering meiosis, suggesting maternal undernutrition delayed germ cell development (Rae *et al.* 2001). In the present study, maternal protein restriction did not alter the developmental progress of germ cells since the proportion of the different germ cell markers was similar in both diets. The proportion of the cortex to medulla within an ovary was similarly not different. This suggests that the explanation for the reduced ovarian weight is due to differences in the number of non-germ cells (e.g. pre-granulosa cells and fibroblasts) or amount/composition of connective tissue (i.e. collagen and fibronectin). Indeed, this is a period of active proliferation of pre-granulosa cells, with a 20-fold increase observed between gd40 and gd75 in sheep (Smith *et al.* 2014).

An alternative explanation for the reduced ovarian weight is that the germ cell weight was altered by diet, while their density and number were unaffected. This is supported by our finding that the estimated germ cell weight was reduced by maternal protein restriction (10.1 ± 1.4 mg (control) vs 7.0 ± 0.5 mg (LP)). This was determined by multiplying the percentage area occupied by germ cells in the cortex by the ovarian weight. However, there are several assumptions in this calculation, such as that ovarian weight is principally affected by germ cells. There are indications that nutritional deficiencies have led to ultrastructural changes in mitochondria of the oocyte from the pre-ovulatory follicle (Schutt *et al.* 2019), although changes in weight were not reported. The underlying mechanism by which any reduction in germ cell weight could occur remains unclear, especially since the developmental stages of the germ cells were not different.

### Germ cell proliferation and apoptosis

Gestation day 65 is nearly half-way through the gestation in sheep and is approaching the time of maximal germ cell numbers (approx. gd75), at which point atresia becomes the more dominant process (Smith *et al.* 1993). Thus, it was important that proliferation and apoptotic indices were determined, especially since at later stages of gestation, effects of maternal undernutrition have previously been reported. For example, there was an increase in germ cell number on gd75 (Smith *et al.* 2019) but delayed follicular development on gd110 (Rae *et al.* 2001) and gd135 (Grazul-Bilska *et al.* 2009). The present study showed that approximately 25–30% of germ cells were Ki67 positive at gd65, indicating that intense cell proliferation was occurring. This was clearly more evident in the cortex nearest to the surface epithelium and agrees with previous reports (Sawyer *et al.* 2002). There was no evidence that maternal protein restriction adversely affected the germ cell proliferation rate in contrast to a previous finding in which a 50% maternal feed restriction (i.e. total energy and macronutrient reduction) decreasedfetal germ cell proliferation (Lea *et al.* 2006). Hence, it is uncertain whether this reflects a differentialfetal response to reduced overall feed intake vs reduced protein, or a difference in the methodologies used in the image analysis.

Activated, cleaved caspase 3 is a common effector of apoptosis and was immunolocalised to thefetal ovaries in the current study. On gd65, apoptotic germ cell death was observed alongside germ cell mitosis. However, the apoptotic index was much lower (approx. 4%) compared to the proliferation index. More importantly, maternal protein restriction did not affect the apoptotic index at gd65. Apoptosis is more dominant in laterfetal ovary development with the loss of >75% of germ cells, between days 75 and 90 in sheep (Smith *et al.* 1993). It is, therefore, feasible that the subsequent 'apoptotic-dominant' phase would be more sensitive to dietary challenges. Indeed, at gd110, the expression of pro-apoptotic factor, Bax was increased in primordial follicles of underfed sheep (Lea *et al.* 2006).

### PAS and ovarian vascularisation

The structural scaffold that surrounds the ovigerous cords and separates them from the ovarian stromal cells is likely to play an important supporting role in regulating germ cell development (McNatty *et al.* 2000, Juengel & Smith 2014). To the best of our knowledge, the effect of maternal diet on thefetal ovarian structural framework has not been investigated. Thus, periodic acid-Schiff (PAS) staining was utilised to identify and quantify the basement membrane surrounding the ovigerous cords (Sawyer *et al.* 2002). The spatial pattern and degree of PAS staining were not affected by dietary treatment suggesting that there was no major change to the ovarian structural framework. However, there are dynamic changes in the expression of structural proteins such as collagen and fibronectin duringfetal ovarian development (Hatzirodos *et al.* 2019) and further investigation into the molecular phenotype of the basement membranes is warranted.

The development of the ovarian vasculature via vasculogenesis is an essential component of ovarian morphogenesis and function (Bussolino *et al.* 1997, Mcfee & Cupp 2013). In the present study, the vasculature was evident in both the cortex as a microvasculature surrounding the ovigerous cords and in the medulla as larger capillaries and/or defined blood vessels. This is in agreement with previous observations (Grazul-Bilska *et al.* 2009, Mcfee & Cupp 2013). The degree of vascularisation in the microvascularised cortex and the vascular beds of the medulla was unaffected by maternal protein restriction on gd65. This would suggest that the supply of nutrients and oxygen to the developing germ cells would be similar in both the control and low protein groups. Intriguingly, in the same fetuses, the microvasculature (identified using CD34 immunohistochemistry) of thefetal kidney was adversely affected by the low protein diet, but the macrovasculature (as measured by vascular corrosion cast) was not (Dunford *et al.* 2014). At this stage of gestation, the kidney is much larger in size than the ovary, making it possible to do vascular corrosion casting to obtain vascular volumetric data (Dunford *et al.* 2014). Similar to the present report, Grazul-Bilska *et al.* (2009) reported no effect of a 60% feed/energy restriction on the ovarian vasculature on gd135. Although, increased endothelial cell expression of pro-apoptotic Bax was reported in underfed sheep on gd110 (Lea *et al.* 2006).Thus, it is feasible that the ovarian vascular development is less sensitive to dietary protein restriction than thefetal kidney.

A key feature of this study is that the extensive characterisation of other organs was conducted on the same protein-restricted sheep. This demonstrated that in addition to the kidney, aspects of liver function were also affected by maternal diet (Dunford *et al.* 2014). This emphasises that the detrimental effects of undernutrition can affect organs differently, perhaps due to differences in ontogeny of organs during developmental; for example, heart developing early, and lungs later. Furthermore, it is tempting to speculate that the lack of effect of maternal protein restriction on the developmental progress of germ cells and ovarian vascularisation represents a process whereby the aspects of ovary are somewhat protected from metabolic stresses. Part of this mechanism might be that vasculogenesis/angiogenesis is regulated differently in the ovary compared with other organs, such that ovarian endothelial cells are less sensitive to metabolic insults. For example, it is known thatfetal vascularisation of the testes and ovary have different mechanisms and regulation (Mcfee & Cupp 2013). This would enable the developingfetal ovary to receive continual nutritional and hormonal support for appropriate development of the germ cells.

In conclusion, on gd65, the ovarian structure and vascularisation, as well as total number of germ cells and their rates of proliferation and apoptosis were not influenced by maternal protein restriction. However,fetal ovarian weight was adversely affected by maternal dietary protein restriction in sheep, while the germ cell count and density were not. In summary, this suggests that a reduced maternal protein has the potential to regulate ovarian development in offspring.

## Declarations of interest

The authors declare that there is no conflict of interest that could be perceived as prejudicing the impartiality of the research reported.

## Funding

This work was supported by the Tertiary Education Trust Fund http://dx.doi.org/10.13039/501100008895 (Tetfund), Nigeria; a British Heart Foundationhttp://dx.doi.org/10.13039/501100000274 PhD studentship (FS/09/011/26562); and a Society for Endocrinology http://dx.doi.org/10.13039/501100000382 Early Career Grant.

## Author contribution statement

C N was involved in investigation, visualization, writing – original draft preparation, funding acquisition. K W was involved in supervision, writing – original draft preparation, writing – reviewing and editing. N B involved in investigation. D G was involved in conceptualization, methodology, resources, funding acquisition. R R was involved in conceptualization, methodology, supervision, formal analysis, visualization, writing – original draft preparation, writing – reviewing and editing
